# Effectiveness of Mannitol Use on Clinical Outcomes of Severe Traumatic Brain Injury Patients

**DOI:** 10.12688/f1000research.148102.1

**Published:** 2024-05-28

**Authors:** Syahrul Syahrul, Nasrul Musadir, Hidayaturrahmi Hidayaturrahmi, Taufik Suryadi, Aqil Naufal Syahrul

**Affiliations:** 1 Neurology, Universitas Syiah Kuala, Banda Aceh, Aceh, Indonesia; 2 Forensic Medicine, Universitas Syiah Kuala, Banda Aceh, Aceh, Indonesia; 3 Universitas Airlangga, Surabaya, East Java, Indonesia

**Keywords:** GODS, Mannitol, Clinical Outcome, Severe Traumatic Brain Injury

## Abstract

**Background:**

Head injuries are considered as a silent epidemic due to the high incidence rate throughout the world. The main cause of morbidity and mortality in patients with head injury is cerebral edema which is defined as abnormal fluid accumulation in the brain parenchyma. Mannitol is a hyperosmolar solution given to reduce fluid volume in the brain. Increased high intracranial pressure can affect prognosis and can be evaluated by assessing clinical outcomes in patients with severe traumatic brain injury using the Glasgow Outcome Discharge Scale (GODS) instrument.

**Methods:**

Observational analytical study with a cross sectional design on 50 patients with severe traumatic brain injury at dr. Zainoel Abidin General Hospital Banda Aceh to determine the effect of mannitol use on the clinical outcomes of severe traumatic brain injury patients which used t test analysis.

**Results:**

The mean value of the group that received mannitol had a higher GODS score than the group that did not receive mannitol. The results of the T test between groups obtained a p value of 0.000 which is smaller than 0.05, so it can be concluded that the use of mannitol has an effect on the GODS score in Severe traumatic brain injury patients. The results showed that the mean GODS value in patients who received mannitol was higher than those who did not receive mannitol.

**Conclusion:**

This concludes that the administration of mannitol is effective in improving the clinical outcomes of patients with severe traumatic brain injury at dr. Zainoel Abidin General Hospital Banda Aceh.

## Introduction

Head injury or traumatic brain injury is structural trauma or functional disorders that affect the brain through external mechanisms. Head injuries are a serious health problem experienced throughout the world because they can cause temporary to permanent impairment in physical, cognitive and psychosocial functions. The severity of head injury is classified based on the level of consciousness using the Glasgow Coma Scale (GCS), namely mild head injury (GCS=13-15), moderate head injury (9-12), and severe head injury (3-8). This classification is also supplemented by assessment of head CT scan findings. ^1^^,^^2^

Head injuries are known as a silent epidemic because their incidence is quite high throughout the world. The global incidence of head injuries is 939 cases per 100,000 population, which means an estimated 69 million people experience head injuries every year. Based on regional divisions according to the World Health Organization (WHO), the highest number of head injury cases occurred in the United States, namely 1299 cases per 100,000 population, followed by Europe with 1012 per 100,000 population, and Southeast Asia with 948 per 100,000 population. Most studies on head injuries show that the incidence of head injuries is twice as common in men as in women. The age range for head injury sufferers is the productive age, namely 29 to 45 years. Recent research reports that head injury incidents also occur at ages 50 to 75 years. The mechanisms causing head injuries can vary based on age range, gender and socio-economic factors. Traffic accidents are the highest cause of head injuries, followed by falls, violence, sports accidents and recreation. ^1^^–^^3^

Research conducted by Syahrul et al. in 2020 at Dr. Zainoel Abidin General Hospital Banda Aceh found that out of 60 head trauma cases, 35 men and 25 women, with an average age of 18-39 years, 42% of patients experienced post-traumatic amnesia. The cause of trauma is 90% of traffic accidents using motorbikes, based on the Glasgow Coma Scale (GCS) assessment, 85% of cases have a GCS score > 9 and 25% with a score < 8, based on the results of head CT scans found in 25% of cases of cerebral edema, 18% of Epidural Hemorrhage, 15% intracranial Hemorrhage and 12% Subarachnoid Hemorrhage, while based on laboratory assessment 80% there was an increase in leukocytes with an increase in neutrophil segments 72%, Hyperglycemia &%, hyponatremia 2%, hypernatremia 11%, hypokalemia 22%, hyperkalemia 17% and hyperchloremia 30%. ^4^

The World Health Organization (WHO) stated that in 2020, head injuries were one of the highest causes of death and disability throughout the world. Deaths due to head injuries were reported in 4.5 million cases or equivalent to 1:10 of all deaths. The mortality rate for severe head injuries ranges from 15–30% within 14 to 30 days after injury. Meanwhile, the significant recovery rate for patients with serious head injuries is also high, with the benchmark being that patients can care for themselves until they can return to work or return to school. ^3^^,^^4^

The main cause of morbidity and mortality in head injury patients is cerebral edema. ^5^ Cerebral edema is defined as an abnormal accumulation of fluid in the brain parenchyma. ^6^ Cerebral edema begins with damage to cells and blood vessels, then followed by activation of cascade injury. The cascade begins with the release of glutamate into the extracellular space, then opens calcium and sodium channels in the cell membrane due to glutamate stimulation. The ATPase pump membrane removes one calcium ion and is replaced with three sodium ions. Sodium in cells creates an osmotic gradient and increases cell volume with the influx of water. ^5^

The Monro-Kellie theory explains cerebral edema in the simplest terms. This theory states that the total volume in the skull bones is three, namely brain tissue 1400 mL (80%), Cerebrospinal Fluid (CSF) 150 mL (10%) and Cerebral Blood Flow (CBF) arteries and veins 150 mL (10%) Bones The skull is a rigid structure, stiff and unable to stretch, which means the volume inside the skull bone is always constant. If the volume of one component increases, it will force a reduction in the volume of the other component. ^7^

Therapeutic management of cerebral edema begins with general measures (optimizing head and neck position to facilitate intracranial venous outflow, avoiding dehydration and systemic hypotension, and maintaining normothermia). The main goals of this therapeutic management are to optimize cerebral perfusion, oxygenation, venous drainage, minimize cerebral metabolism and avoid ionic disturbances or gradients between the brain and vascular compartments. ^8^

Mannitol is a hyperosmolar solution given to reduce fluid volume in the brain. ^9^ There are two main mechanisms for mannitol to reduce intracranial hemorrhage ICH. The first mechanism is to increase the osmotic gradient of the brain barrier, where molecules are not free to diffuse (low permeability coefficient). Mannitol causes osmosis of water from the brain parenchyma, resulting in a decrease in brain water content and an increase in extracellular volume. Reducing brain water content reduces perilesional edema, which has been demonstrated in several clinical trials and animal tests. The second mechanism is related to rheological effects, mannitol can reduce hematocrit, viscosity and deformability of red blood cells resulting in increased microvascular flow and increased cardiac output and Mean Arterial Pressure (MAP). Increased blood and oxygen flow to the brain as well as cerebral vasoconstriction further reduce cerebral blood volume, reducing ICP and increasing CPP. ^10^

The dose of mannitol for the treatment of cerebral edema is 0.25-1 g/kg. Loading dose (LD) 1g/kg BW, followed by maintenance dose (MD) 0.5 6 g/kg BW every 4-6 hours. The half-life of mannitol is 2.2-2.4 hours. Efficacy is visible within 15-30 minutes, and the duration of effect is 90 minutes to 6 hours. The mannittol preparation used in this study was 1 g/kgBW where 1 g was equivalent to 5 cc as the initial dose given for 30 minutes, 6 hours later given 0.25 g/kgBW which was reduced gradually over 3 days to prevent rebound phenomena. Mannittol preparations are available at hospital pharmacy installations as hyperosmolar liquid with batch number A50J52A with a supplier from Otsuka. Mannitol administration is recommended as a bolus and not as a continuous infusion in this therapy to maximize benefits and minimize side effects although there are no direct clinical trials comparing these two administration techniques. ^10^

The prognosis of head injury patients is something that every clinician must pay attention to. The patient's family often asks the doctor questions regarding the patient's prognosis, such as the patient's awareness after injury, the possibility that the patient will be able to return to work or return to school. Information that can be used as a predictor of prognosis in patients with head injuries is very useful for clinicians in communicating with patients and families, making therapeutic decisions, and utilizing health personnel resources in treating patients with head injuries. ^11^^,^^12^

To assess the outcome of head injury patients, you can use the Glasgow Outcome Scale (GOS) instrument, which is the most widely used measurement scale. The Glasgow Outcome Scale (GOS) has been developed into the Glasgow Outcome Scale Extended (GOSE), adopting a standard format for the interviews used to determine outcomes. Another outcome assessment scale for head injury patients issued by the University of Glasgow is the Glasgow Outcome at Discharge Scale (GODS) which is assessed when the patient has finished treatment. The GODS scale was proven to be valid and reliable for assessing the outcome of head injury patients. ^1^^–^^3^

Based on the description above, the condition of cerebral edema in severe traumatic brain injury needs to be treated by administering mannitol to reduce ICP so that the patient's clinical outcome will be better. Therefore, this study wants to analyze the effectiveness of using mannitol on the clinical outcomes of patients with severe traumatic brain injury using the Glasgow Outcome at Discharge Scale (GODS) outcome scale at the end of treatment at the dr. Zainoel Abidin General Hospital (ZAH) Banda Aceh.

## Methods

This research uses an analytical research design with a cross-sectional approach (cross sectional study). This research was conducted at ZAH. The population in this study were all head injury patients at ZAH in June-August 2023. The sample for this study was some head injury patients in the neurology inpatient ward at ZAH who meets the specified inclusion and exclusion criteria. The sample criteria used were age over 18 years, diagnosed with a serious head injury, onset of head injury ≤ 24 hours, and a CT scan of the head without contrast. This study also has exclusion criteria, namely patients who experienced multiple trauma, patients who were found to have non-traumatic abnormalities on a CT scan of the head, patients with a history of diabetes mellitus or cardiovascular disorders before injury, patients suffering from malignant diseases (neoplasms), patients who underwent surgical operations neurology, and Patients Returning Home at Their Own Request. The sample size and sampling technique in this study were calculated and the sample size was 47 patients.

The sampling technique was non-probability sampling with consecutive sampling. For samples who were unable to carry out interviews, those who acted as research respondents were companions. The patient's companion is the person who accompanies the patient during the treatment period in the inpatient room and is the one who knows the patient's progress best until the patient has finished undergoing treatment.

### Data collection

The data sources in this study were obtained from primary data and secondary data, primary data was used to assess interview results to determine the GODS score, while secondary data was obtained from medical records to investigate the administration of mannitol. The GODS instrument is a questionnaire formula for assessing the outcome of head injury patients, and is carried out when the patient has finished hospitalization. The GODS instrument was developed by the University of Glasgow in 2012. The GODS has proven to be very useful for research purposes or other clinical purposes. GODS can be used routinely by every medical worker who cares for patients with head injuries to assess PTA, attention and memory. The GODS focuses on disability, so that GODS can be used accurately and quickly by medical staff to make decisions about outpatient care (discharge planning).

The GODS instrument was prepared in English and to date has never been translated into Indonesian. In this study, the research will adapt the GODS questionnaire. The process of adapting the questionnaire begins with the translation stage, namely translating the GODS questionnaire into Indonesian. The questionnaire was then translated back into English by another translator. Then, the results of the translation in English are compared with the original questionnaire, assessing whether there is a change in meaning or not.

The research began by recording head injury patients who were admitted to the ZAH emergency room within ≤24 hours after the injury, then taking research samples that met the inclusion and exclusion criteria. The researcher then gave informed consent to the patient or the patient's companion. Giving informed consent was done in writing and was approved by the research ethics committee. Researchers collected data on the use of mannitol in patients with severe head injuries. Researchers assessed patient outcomes using the GODS form which was carried out when the patient was declared to have finished inpatient treatment. All data was recorded on a research sheet and analyzed univariately and bivariately.

### Statistical analysis

Univariate analysis is used to analyze each research variable with the aim of assessing the percentage distribution of the variables observed descriptively. Bivariate data analysis to determine the effect of mannitol use on the clinical outcomes of severe traumatic brain injury patients at ZAH using the independent T-test with a significance value of 0.05 or a confidence level of 95%.

### Ethics approval

This study was approved by the Health Research Ethics Committee of the ZAH, Indonesia, No. 133/ETIK-RSUDZA/2023. Research ethical approval was issued on June 9, 2023.

## Result

This research was carried out from June 2023 to August 2023. The sample for this study was 50 patients with severe traumatic brain injury treated at ZAH, divided into 2 groups of patients who received mannitol and those who did not receive mannitol therapy, as well as interviews to assess the outcomes of head injury patients using the GODS scale. The subject characteristic can be seen in Table 1.

**Table 1.** Sample Characteristics of Severe Traumatic Brain Injury Patients at Dr. Zainoel Abidin General Hospital Banda Aceh.

Characteristic	Frequency	Percentage (%)
**Gender**		
Female	15	30
Male	35	70
**Total**	**50**	**100**
**Age (Years)**		
18 – 31	23	46
32 – 45	12	24
46 – 60	11	22
> 60	4	8
**Total**	**50**	**100**
**Head CT Scan Results**		
**Lesion Bleeding**		
Intracerebral hemorrhage	5	10
Subarachnoid Hemorrhage	10	20
Epidural Bleeding	5	10
Multiple bleeding lesions	28	56
**Lesion Without Bleeding**		
Cerebral Edema	2	4
**Total**	50	**100**

Source: Primary Data, 2023.

Based on this research, it was found that the incidence of Severe traumatic brain injury occurred more frequently in male, namely 70%, based on the age group, the majority was found at the age of 18-30 years, namely 46%. Based on the lesion findings on head CT scans in Severe traumatic brain injury, it is a bleeding lesion multiple as much as 56%. The results of this study showed 50 Severe traumatic brain injury patients consisting of 35 male patients (70%) and 15 female patients (30%).

Based on the findings of intracranial hemorrhage lesions, 48 patients were found with intracranial hemorrhage lesions in the form of subarachnoid hemorrhage in 10 patients (20%), intracerebral hemorrhage in 5 patients (10%), epidural hemorrhage in 5 patients (10%) and multiple hemorrhages were found in 28 patients (56%). Overall, the results of this study show that a CT scan of the head can identify primary head injuries or secondary head injuries in the form of cerebral edema.

Distribution of GODS results for patients with severe traumatic brain injury can be seen in Table 2.

**Table 2.** Distribution of GODS results for patients with severe traumatic brain injury at ZAH.

GODS score	Frequency (N)	Percentage (%)
1 = Died	9	18%
2 = Unconscious	0	0%
3 = Lower Severe Disability	1	2%
4 = Upper Severe Disability	3	6%
5 = Lower Moderate Disability	4	8%
6 = Upper Moderate Disability	8	16%
7 = Lower Good Recovery	14	28%
8 = Upper Good Recovery	11	22%
**Total**	**50**	**100%**

Source: Primary Data, 2023.

The results of the study showed that there were 9 patients who died during the treatment period after Severe traumatic brain injury (18%). Patients with good outcomes (Good Recovery) were found in the majority of patients, with details of 11 patients showing Upper Good Recovery (22%) and 14 patients showing Lower Good Recovery outcomes (28%). GODS Results for Severe Traumatic Brain Injury Patients at dr. Zainoel Abidin General Hospital Banda Aceh can be seen in Table 3.

**Table 3.** GODS results for severe traumatic brain injury patients at Dr. Zainoel Abidin General Hospital Banda Aceh.

Variable	Mean	Standard Deviation	Median	Minimum	Maximum	Normality test (p)
GODS score	5.56	2.47	6.50	1	8	<0.005

Source: Primary Data, 2023.

The research results show the mean GODS score is 5.56 and the median is 6.50. The lowest GODS value is 1, and the highest value is 8 with a standard deviation of 2.47. The results of the study showed that there were 9 patients who died during the treatment period after head injury (18%). Patients with good outcomes (Good Recovery) were found in the majority of patients. The relationship between mannitol administration and GODS scores in patients with severe traumatic brain injury at dr. Zainoel Abidin General Hospital Banda Aceh can be seen in Table 4.

**Table 4.** The relationship between mannitol administration and GODS scores in patients with severe traumatic brain injury at Dr. Zainoel Abidin General Hospital Banda Aceh.

Mannitol	N	Mean	Standard Deviation	St. Error	P. Value
Yes	25	6.88	1.6	0.3	0.000
No	25	4.24	2.48	0.49	

Based on Table 4, it was found that the mean value of the group that received mannitol had a higher GODS score than the group that did not receive mannitol. The results of the T test between groups obtained a p value of 0.000 which is smaller than 0.05, so it can be concluded that the use of mannitol has an effect on the GODS score in Severe traumatic brain injury patients.

## Discussion

The present study are in accordance with the epidemiology of head injuries in Europe which shows that the ratio of the incidence of head injuries compared to women ranges from 1.2:1 to 4.6:1. ^13^ The results of this study are also in accordance with research on head injuries in Indonesia. For example, research conducted at Hasan Sadikin Hospital in Bandung in 2008-2010 showed that head injuries occurred more frequently in men. As well as the latest research conducted at M DJamil Hospital Padang in 2020, which shows that head injuries occur more frequently in men than women with a ratio of around 70%: 30%. ^14^^,^^15^

The present study show that based on age group, head injuries most often occur in the productive age group, namely in the 18-30 year age group (46%), followed by the 32-45 year age group (24%), and the 46-60 year age group (22%). These results are in accordance with Imran's 2017 research at ZAH showing that the average age of head injury patients is 30 years. ^16^ Research at Hasan Sadikin Hospital in Bandung also shows that head injuries occur more frequently in the productive age group (aged 18-45 years). ^14^

Overall, the present study in ZAH, show that head injury patients are in accordance with the epidemiology of head injuries throughout the world and in Indonesia, namely that cases occur more frequently in men than women, and often occur in those of productive age. This can be caused by the higher mobility and productivity of men and the productive age group compared to other age groups.

The present study also show that it is most likely that patients who experience intracranial bleeding after a head injury have multiple bleeding. This is in accordance with the theory that intracranial bleeding in head injuries is a primary brain injury due to a direct injury mechanism resulting from the trauma that occurred, and can occur in the coup area or counter-coup area as a result of shock to the brain tissue that occurs and rubs against the skull bone. Bleeding that occurs in all layers of the brain, causing bleeding in the form of hematoma or epidural, submissile or intracerebral bleeding. ^17^^,^^18^ Head CT scan results that did not show intracerebral hemorrhage lesions were found in 2 patients, namely cerebral edema. This is in accordance with research conducted by Khairunnisa in 2020 at ZAH which showed that a normal CT scan of the head or cerebral edema could be found in head injury patients. ^19^

The present study show that there is a possibility that patients will experience diffuse edema lesions due to head injury even without focal lesions of intracranial hemorrhage. This is in accordance with the theory that the pathophysiology underlying cerebral edema is neuroinflammation which can cause damage to the blood brain barrier (BBB) in the first 24 hours after head injury. This BBB damage results in infiltration of neutrophils, monocytes and lymphocytes into the injured brain parenchyma, resulting in polymorphonuclear leukocytes releasing complement factors and pro-inflammatory cytokines such as IL-1b, IL-6 and TNF-a at 24 hours post-trauma and resulting in changes in barrier permeability. brain blood, edema formation, and neurological deficits. ^20^^–^^22^ The present study are in accordance with research conducted by Oliveira et al. In 2012, the GODS scale was used to assess the outcomes of head injury patients, and the GODS scale can assess all outcomes of head injury patients in detail, starting from death, vegetative state to good recovery. ^23^

The GODS instrument was created by the University of Glasgow in 2012, which is a questionnaire to assess the outcome of head injury patients, and is carried out when the patient has finished hospitalization. GODS has proven to be very useful for research or other clinical purposes. GODS can be used routinely by every medical worker who cares for patients with head injuries to assess PTA, attention and memory. The GODS instrument focuses on disability rather than damage, so that GODS can be used accurately and quickly by medical staff to make decisions about outpatient care (discharge planning). The GODS assessment results were also shown to be in accordance with GOSE assessed at 3, 6 or 12 months after head injury. ^23^^–^^26^

The present study showed that 9 head injury patients died (18%). These results indicate that there is a high probability that patients admitted with head injuries to ZAH will be in serious condition. Thus, the readiness of medical personnel in various fields, and the management program for head injury patients must always be ensured in good condition. The results of this study also show that the GODS instrument can be applied at ZAH to assess the outcomes of head injury patients treated at ZAH. The results of the GODS assessment can provide additional information for clinicians to educate patients and families regarding the patient's condition after inpatient treatment, and develop an outpatient program for patients when they return to their pre-head injury activities. This is very important and meaningful, especially for patients considering that ZAH is the main referral hospital in Aceh Province.

Mannitol is an osmotic diuretic used in emergencies and neurosurgery to treat increased intracranial pressure, which can occur after head trauma, neurosurgery, and other types of intracranial pathology. Mannitol is used to reduce increased intracranial pressure (ICP). The available preparation is liquid mannitol with a concentration of 20% (20 grams per 100 ml of solution). Mannitol can be given as an intravenous bolus or through a continuous infusion. The initial bolus dose usually given is 0.5-1.0 g/kg IV. Subsequent doses vary greatly. ^27^^–^^29^

Brain damage due to head injury causes an increase in intracranial pressure which can worsen patient outcomes which can be evaluated using the GODS score. Cerebral edema is a frequent and challenging problem in clinics and is a major cause of morbidity and mortality in brain injury patients. ^8^ Cerebral edema with increased ICP occurs in most secondary brain injuries. ^27^ Cerebral edema is defined as an abnormal accumulation of fluid in the brain parenchyma. ^6^ Cerebral edema begins with damage to cells and blood vessels, then followed by activation of cascade injury. The cascade is initiated by the release of glutamate into the extracellular space. Calcium and sodium channels in cell membranes are opened by glutamate stimulation. The ATPase pump membrane removes one calcium ion and is replaced with three sodium ions. Sodium in cells creates an osmotic gradient and increases cell volume with the influx of water. ^9^

Based on this study, it was found that there were better GODS scores for patients who received mannitol therapy compared to patients who did not receive mannitol. The group that did not receive mannitol was because they did not meet the criteria for receiving mannitol, namely poor kidney and heart function. Mannitol is highly irritating to veins. When administering mannitol, you must use a filtered needle because crystals can form in the solution and be carried into the injection if you are not careful. In addition, monitoring laboratory test results and careful recording of intake and output to assess fluid volume status due to diuresis can be very important. High doses of mannitol have a risk of developing acute renal failure, especially in serum osmolarity > 320 mOsm/L, use of other nephrotoxic drugs, sepsis, previous kidney disease. Another side effect of mannitol is the rebound phenomenon, namely an increase in intracranial pressure when using mannitol for a long time, resulting in the accumulation of mannitol in the brain tissue so that the osmotic pressure becomes higher in the tissue which attracts fluid from the blood vessels to the brain tissue which causes brain edema heavier. ^30^

The new finding from this study is that the GODS instrument can be used to assess the outcomes of head injury patients at ZAH. The GODS instrument is an instrument in the form of a detailed and detailed list of questions to assess the outcomes of head injury patients proposed by the University of Glasgow, which is an institution that also introduced the Glasgow Outcome Scale (GOS) and Glasgow Outcome Scale Extended (GOSE). The difference between GODS and GOS or GOSE is that GODS aims to assess the patient's disability after a head injury while the patient is still being treated and is planned for outpatient care (discharge planning). The GODS instrument has proven to be very useful for research purposes or other clinical purposes. The GODS instrument can be used routinely by every medical worker who cares for patients with head injuries to assess PTA, attention and memory. The GODS instrument focuses on disability rather than damage, so that GODS can be used accurately and quickly by medical staff to make decisions about outpatient treatment (discharge planning). Easily assessing the degree of disability of head injury patients is very useful for patients.

The present study as a whole show that treatment of patients with severe traumatic brain injury must be carried out appropriately and quickly. Therapeutic management of head injuries using mannitol should be carried out within the first ≤24 hours. Clinicians can then assess the outcome of head injury patients using the GODS instrument which is assessed when the patient has finished hospitalization.

## Conclusions

Based on the results of the research and discussion previously explained, it can be concluded that the characteristics of head injury patients at ZAH are in accordance with the epidemiology of head injuries, namely that they occur more frequently in men than women. Head injuries have also been proven to occur most frequently in the productive age group. Administration of mannitol is effective in improving the clinical outcomes of patients with severe traumatic brain injury at ZAH if assessed using the GODS instrument.

## Consent to publish

We have obtained written consent to publish from each individual respondent when the respondent filled out their consent to participate in this research. In the informed consent form it was stated that the results of this research would be published in a reputable international journal and also stated in the research protocol.

## Ethics and consent

This study was approved by the Health Research Ethics Committee of the ZAH, Indonesia, No. 133/ETIK-RSUDZA/2023 June 9, 2023.

## Data Availability

Zenodo, Effectiveness of Mannitol Use on Clinical Outcomes of Severe Traumatic Brain Injury Patients https://zenodo.org/records/10983092. ^31^ This project contains the following underlying data: • Master data for effectiveness of mannitol.xls. Data are available under the terms of the Creative Commons Attribution 4.0 International license (CC-BY 4.0). AGREE Reporting Guideline Checklist is available on Zenodo: https://zenodo.org/records/11091191. ^32^ Zenodo: Extended data: Effectiveness of Mannitol Use on Clinical Outcomes of Severe Traumatic Brain Injury Patients, https://doi.org/10.5281/zenodo.11113667. ^33^ Data are available under the terms of the Creative Commons Attribution 4.0 International license (CC-BY 4.0).
