# Supplementary material for: Effectiveness of Mannitol Use on Clinical Outcomes of Severe Traumatic Brain Injury Patients
Source: F1000Res. 2024 May 28;13:548. [Version 1] doi: 10.12688/f1000research.148102.1 (PMC11393529; doi:10.12688/f1000research.148102.1)
Supplement: Peer review history

# Reviewer report: Reviewer response for version 1

**DOI:** 10.5256/f1000research.162371.r316006

**Published:** 2024-09-12

**Authors:** Muhammad Ashir Shafique

**Affiliations:** 1 Jinnah Sindh Medical University, Karachi, Pakistan

This paper investigates the impact of mannitol administration on clinical outcomes for severe traumatic brain injury (TBI) patients using the Glasgow Outcome at Discharge Scale (GODS). The study is carried out at Dr. Zainoel Abidin General Hospital (ZAH), Banda Aceh, Indonesia, between June and August 2023. It focuses on determining whether mannitol treatment can effectively improve clinical outcomes for patients with severe TBI, assessed using GODS. The study design is cross-sectional and employs both primary and secondary data collection. It examines 50 patients divided into two groups: those who received mannitol and those who did not.

 - The paper does not discuss recent meta-analyses or systematic reviews on mannitol efficacy in severe TBI. Incorporating a comparison with findings from the two current meta-analyses ( [ ref 1 ] and [ ref 2 ] ) would provide additional context and validation.

 - The sampling technique used is "non-probability sampling with consecutive sampling," which may introduce bias. A randomized sampling method would improve the robustness of the study and its generalizability.

 - There is insufficient detail about the characteristics of the control group (those who did not receive mannitol). A comparative analysis of baseline characteristics between the mannitol and control groups is needed.

 - Table 4 oversimplifies the relationship between mannitol administration and GODS scores. A multivariate analysis should be used to adjust for confounding factors such as initial GCS score, age, and comorbidities.

 - The study lacks a power analysis to determine if the sample size is adequate. Conducting a power analysis would help ensure the study is sufficiently powered to detect significant effects.

 - Terminology such as "cerebral edema," "intracranial hemorrhage," and "severe traumatic brain injury" is used inconsistently. Clarifying and standardizing these terms throughout the manuscript is important.

 - Although the GODS instrument was translated into Indonesian, there is no validation of this translation. Providing evidence of validation for the translated GODS instrument is necessary.

 - The study only assesses outcomes at discharge and does not include long-term recovery or complications. Including a follow-up period would provide insight into the long-term efficacy of mannitol.

 - The statistical methods are not clearly described, particularly regarding the T-test application. A more detailed explanation of the statistical analyses used, including assumptions for the T-test, is needed.

 - Conducted at a single center, the study's generalizability may be limited. Discussing the limitations related to generalizability and considering data from multiple centers could enhance the external validity of the findings.

Is the work clearly and accurately presented and does it cite the current literature?

No

If applicable, is the statistical analysis and its interpretation appropriate?

No

Are all the source data underlying the results available to ensure full reproducibility?

Yes

Is the study design appropriate and is the work technically sound?

Yes

Are the conclusions drawn adequately supported by the results?

Yes

Are sufficient details of methods and analysis provided to allow replication by others?

Yes

Reviewer Expertise:

The curretn haven't done a proper and complete lit review

I confirm that I have read this submission and believe that I have an appropriate level of expertise to confirm that it is of an acceptable scientific standard, however I have significant reservations, as outlined above.

## References

[1] : Efficacy of hypertonic saline and mannitol in patients with traumatic brain injury and cerebral edema: a systematic review and meta-analysis. *Egyptian Journal of Neurosurgery* .2023;38(1) : 10.1186/s41984-023-00233-w 10.1186/s41984-023-00233-w

[2] : Comparing the effects of mannitol and hypertonic saline in severe traumatic brain injury patients with elevated intracranial pressure: a systematic review and meta-analysis. *Neurol Res* .2024;46(9) : 10.1080/01616412.2024.2360862 883-892 10.1080/01616412.2024.2360862 38825027

# Reviewer report: Reviewer response for version 1

**DOI:** 10.5256/f1000research.162371.r296717

**Published:** 2024-08-30

**Authors:** Farhad Bal’afif

**Affiliations:** 1 Department of Surgery, Brawijaya University/Saiful Anwar Hospital Malang, Malang, Indonesia

The article investigates the effectiveness of mannitol in improving clinical outcomes for patients with severe traumatic brain injury (TBI) through an observational analytical study at dr. Zainoel Abidin General Hospital in Banda Aceh. The study included 50 patients, comparing those who received mannitol therapy with those who did not, using the Glasgow Outcome Discharge Scale (GODS) to assess outcomes. Key findings indicate that patients who received mannitol had significantly higher GODS scores, suggesting improved recovery outcomes. However, the study's cross-sectional design, though suitable for initial observations, lacks longitudinal follow-up, which could provide deeper insights into long-term patient outcomes. Despite these limitations, the findings support mannitol potential in enhancing recovery for severe TBI patients, highlighting avenues for future research to explore its comparative effectiveness and broader applicability in clinical practice.

Is the work clearly and accurately presented and does it cite the current literature?

Yes

If applicable, is the statistical analysis and its interpretation appropriate?

Yes

Are all the source data underlying the results available to ensure full reproducibility?

Yes

Is the study design appropriate and is the work technically sound?

Yes

Are the conclusions drawn adequately supported by the results?

Yes

Are sufficient details of methods and analysis provided to allow replication by others?

Yes

Reviewer Expertise:

Neurosurgery focus on vp shunt

I confirm that I have read this submission and believe that I have an appropriate level of expertise to confirm that it is of an acceptable scientific standard.

# Reviewer report: Reviewer response for version 1

**DOI:** 10.5256/f1000research.162371.r296718

**Published:** 2024-07-24

**Authors:** Brandon Lucke-Wold

**Affiliations:** 1 Lillian S. Wells Department of Neurosurgery, University of Florida, Gainesville, Florida, USA

Overall mannitol offers an acute benefit for severe brain injury. The paper looks at systemic review for individual medication. In clinical scenarios, mannitol and hypertonic saline are often used in conjunction. 

 The authors need to address this possibility of confounding and other medications that may be influencing the reported outcomes. 

 Interesting *paper looking at *mannitol for TBI. 

 Authors should address comparison to hypertonic saline 

- [ref 1] and implications for various imaging concerns [ref2]

If the above are addressed and references included, paper would be of interest. 

 Should also address how country factors can be of importance to overall outcomes.

Is the work clearly and accurately presented and does it cite the current literature?

Yes

If applicable, is the statistical analysis and its interpretation appropriate?

Yes

Are all the source data underlying the results available to ensure full reproducibility?

Yes

Is the study design appropriate and is the work technically sound?

Yes

Are the conclusions drawn adequately supported by the results?

Yes

Are sufficient details of methods and analysis provided to allow replication by others?

Yes

Reviewer Expertise:

Subarachnoid hemorrhage and TBI

I confirm that I have read this submission and believe that I have an appropriate level of expertise to confirm that it is of an acceptable scientific standard, however I have significant reservations, as outlined above.

## References

[1] : Comparing the effects of mannitol and hypertonic saline in severe traumatic brain injury patients with elevated intracranial pressure: a systematic review and meta-analysis. *Neurol Res* .2024; 10.1080/01616412.2024.2360862 1-10 10.1080/01616412.2024.2360862 38825027

[2] : Updates on Improving Imaging Modalities for Traumatic Brain Injury. *J Integr Neurosci* .2023;22(6) : 10.31083/j.jin2206142 142 10.31083/j.jin2206142 38176928
